# Supplementary material for: Temperature dependence of mosquitoes: Comparing mechanistic and machine learning approaches
Source: PLoS Negl Trop Dis. 2024 Sep 16;18(9):e0012488. doi: 10.1371/journal.pntd.0012488 (PMC11460681; doi:10.1371/journal.pntd.0012488)
Supplement: S1 Text — Table A: Filtered number of occurrences by species after each data cleaning step. Table B: Environmental covariates and respective data sources, accessed from Google Earth Engine. Table C: Model performance. Median and range of AUC (min-max) across bootstrapping iterations for both in-sample and out-of-sample performance. Table D: Thermal minima, optima, and maxima with rank ordering across species for the mechanistic trait based model (M(T)) versus the species distribution model (partial dependence plots (PDPs)). Fig A: Pairwise correlation plot of environmental predictors used in the model. A pairwise correlation analysis was run for each set of two covariates, and any variables that had a correlation that exceeded the R < |0.8| threshold were reassessed and modified, or dropped from the analysis entirely. CD is cattle density; EVIM is enhanced vegetation index mean; EVISD is enhanced vegetation index standard deviation; FC is forest cover percentage; HPD is human population density; PDQ is precipitation of the driest quarter; PhotoASTM is photoperiod activity season temperature annual mean; PhotoASTSD is photoperiod activity season temperature standard deviation; PrecipASTM is precipitation activity season temperature annual mean; PrecipASTSD is precipitation activity season temperature standard deviation; PWQ is precipitation of the wettest quarter; SW is surface water seasonality; TAM is year-round temperature annual mean; TASD is year-round temperature annual standard deviation; ARH is average relative humidity; and WS is wind speed. There are NAs among temperature variables as there was only one set included in each model (e.g., the Ae. albopictus model included PhotoASTM and PhotoASTD but not TAM, TASD, PrecipASTM, or PrecipASTD). Fig B: Photoperiod activity season. Map of the world shaded in with the length of the photoperiod activity season. Fig C: Precipitation activity season. Map of Africa shaded in with the length of the precipitation activity season i [file pntd.0012488.s001.pdf]

<u>Supplementary text 1:</u> Temperature dependence of mosquitoes: comparing mechanistic and machine learning approaches

Tejas S. Athni[1,2], Marissa L. Childs[3,4], Caroline K. Glidden[2,5], Erin A. Mordecai[2]

Table A. Filtered number of occurrences by species after each data cleaning step.

Species	*Ae. aegypti*	*Ae. albo-pictus*	*An. gambiae*	*An. stephensi*	*Cx. pipiens*	*Cx. quinque-fasciatus*	*Cx. tarsalis*
**Raw Occurrences**	37,115	39,488	13,650	1,232	98,276	30,978	44,496
**Known Basis of Record**	36,981	39,449	13,650	1,196	98,248	30,964	44,487
**Records Within 2000-2019 Range**	30,956	34,399	10,116	540	60,078	27,823	34,361
**Coordinate Uncertainty Reported**	30,908	34,122	10,116	540	59,661	27,744	34,358
**Coordinate Uncertainty Decimal Filter**	30,767	34,047	10,114	540	59,661	27,738	34,358
**Within Landmass**	30,651	33,885	10,109	535	59,629	27,640	35,358
**Within Activity Season**	30,651	29,750	10,054	535	59,429	27,640	35,348
**Complete Predictors**	30,598	29,714	10,052	534	59,415	27,560	31,969
**Unique Cell Centroids**	9,299	8,688	903	358	1,949	2,670	909

Table B. Environmental covariates and respective data sources, accessed from Google Earth Engine.

Covariate	Acronym	Source	Units	Spatial Resolution †,*	Temporal Resolution ^
Cattle Density	CD	Gridded Livestock of the World v3 [1]	Animals per 1 sq. kilometer	9.25 km #	Single year (2010)
Enhanced Vegetation Index Mean	EVIM	NASA MOD13A2 [2]	EVI	1 km	16 days
Enhanced Vegetation Index Standard Deviation	EVISD	NASA MOD13A2 [2]	EVI	1 km	16 days
Forest Cover	FC	NASA MOD44B [3]	% of a pixel	250 m	Yearly
Human Population Density	HPD	GHS-POP [4]	Persons per 1 sq. kilometer	1 km	Single year (2000, 2015)
Precipitation of the Driest Quarter	PDQ	ERA5 [5]	Millimeters	27.83 km	Daily
Precipitation of the Wettest Quarter	PWQ	ERA5 [5]	Millimeters	27.83 km	Daily
Surface Water Seasonality	SW	JRC Global Surface Water [6]	Number of months	30 m	Single year (2014-15)
Temperature Mean in Photoperiod Activity Season	Photo ASTM	ERA5 [5]	°C	27.83 km	Daily
Temperature Standard Deviation in Photoperiod Activity Season	Photo ASTSD	ERA5 [5]	°C	27.83 km	Daily
Temperature Mean in Precipitation Activity Season	Precip ASTM	ERA5 [5]	°C	27.83 km	Daily
Temperature Standard Deviation in Precipitation Activity Season	Precip ASTSD	ERA5 [5]	°C	27.83 km	Daily
Temperature Mean Year-Round	TAM	ERA5 [5]	°C	27.83 km	Daily
Temperature Standard Deviation Year-Round	TASD	ERA5 [5]	°C	27.83 km	Daily
Wind Speed	WS	TerraClimate [7]	Meters per second	4.64 km	Monthly

Average relative humidity	ARH	ERA5 [5]	%	27.83 km	Daily

† The following conversions are used to acquire spatial resolution in kilometers:
1 minute of arc ≈ 0.0166667 degrees,
1 degree ≈ 111 kilometers
* All rasters are resampled to 1 km x 1 km grid cells before use as a predictor in the machine learning model
^ All rasters are averaged over the 2000-2019 study period, if adequate temporal data is available
# Sourced as 5 arcmin

Table C. Model performance. Median and range of AUC (min-max) across bootstrapping iterations for both in-sample and out-of-sample performance.

Species	In-sample AUC	Out-sample AUC
Ae. aegypti	0.999 (0.9959-0.9999)	0.9887 (0.9864-0.9918)
Ae. albopictus	0.9982 (0.9963-0.9998)	0.984 (0.9815-0.9861)
An. gambiae	0.998 (0.9864-0.9999)	0.9366 (0.9149-0.9613)
An. stephensi	1 (0.9993-1)	0.9411 (0.9066-0.9721)
Cx. pipiens	0.9979 (0.9887-0.9999)	0.9347 (0.9184-0.9461)
Cx. quinquefasciatus	0.9996 (0.9981-0.9999)	0.9882 (0.9839-0.9915)
Cx. tarsalis	0.9967 (0.9881-0.9992)	0.9583 (0.9504-0.9743)

Table D. Thermal minima, optima, and maxima with rank ordering across species for the mechanistic trait based model (M(T)) versus the species distribution model (partial dependence plots (PDPs)).

Species	Model	Thermal Minima (Rank)	Thermal Optima (Rank)	Thermal Maxima (Rank) [#]
Aedes aegypti	M(T)	14.9 °C (3)	27.4 °C (1)	34.4 °C (3)
	PDP	9.8 °C (4)	25.6°C (3)	--
Aedes albopictus	M(T)	13.7 °C (4)	25.5 °C (2)	31.3 °C (7)
	PDP	8.8 °C (5)	23 °C (4)	--
Anopheles gambiae	M(T)	19.6 °C (1)	24.7 °C (3)	32.2 °C (4)
	PDP	18.5 °C (1)	26.5 °C (2)	--
Anopheles stephensi	M(T)	15.4 °C (2)	24.1 °C (4)	37.1 °C (1)
	PDP	15.5 °C (2)	26.9 °C (1)	--
Culex pipiens	M(T)	9.5 °C (6)	22.4 °C (7)	34.8 °C (2)
	PDP	5.5 °C (7)	13 °C (6)	--
Culex quinquefasciatus	M(T)	13.6 °C (5)	23.6 °C (5)	31.7 °C (6)
	PDP	11.1 °C (3)	22°C (5)	--
Culex tarsalis	M(T)	6.9 °C (7)	23 °C (6)	31.9 °C (5)
	PDP	7.2 °C (6)	11.5 °C (7)	--

[#] Thermal maxima were not discernible from the partial dependence plots, and are thus not reported.

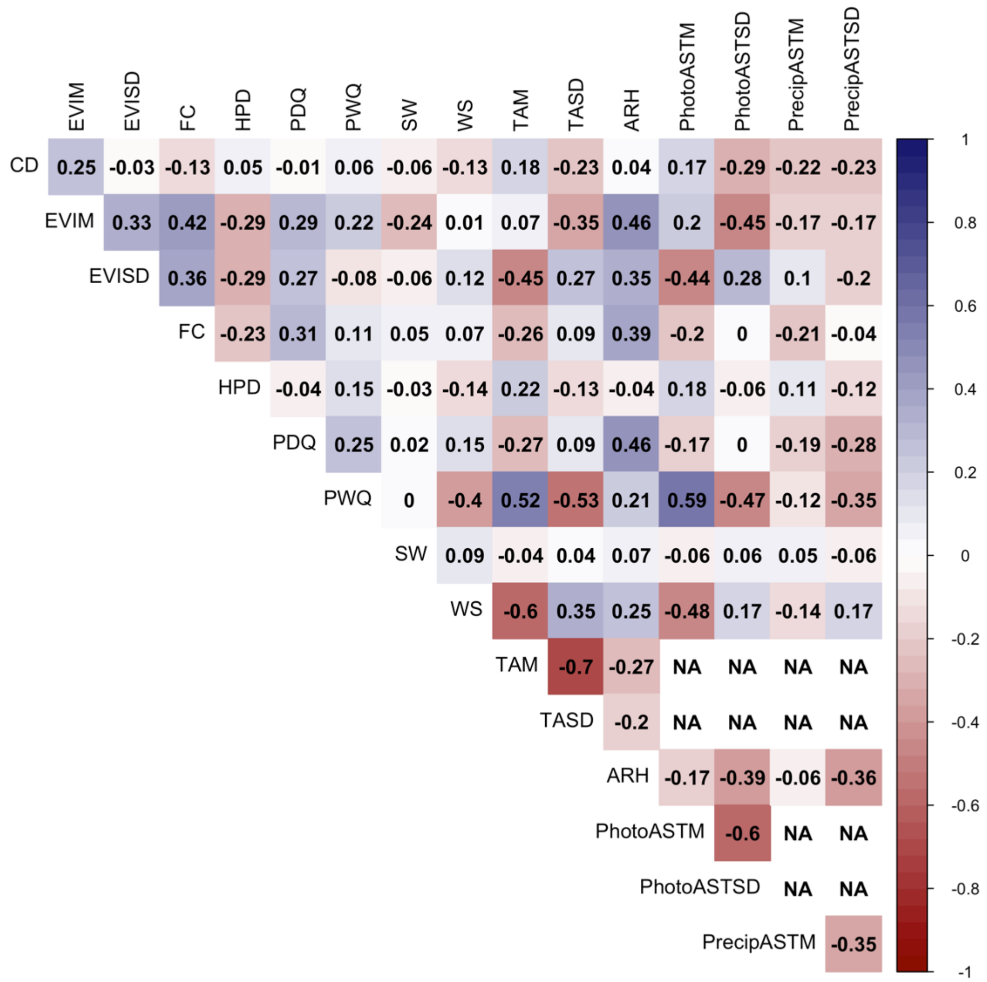

Figure A. Pairwise correlation plot of environmental predictors used in the model. A pairwise correlation analysis was run for each set of two covariates, and any variables that had a correlation that exceeded the R < |0.8| threshold were reassessed and modified, or dropped from the analysis entirely. CD is cattle density; EVIM is enhanced vegetation index mean; EVISD is enhanced vegetation index standard deviation; FC is forest cover percentage; HPD is human population density; PDQ is precipitation of the driest quarter; PhotoASTM is photoperiod activity season temperature annual mean; PhotoASTSD is photoperiod activity season temperature standard deviation; PrecipASTM is precipitation activity season temperature annual mean; PrecipASTSD is precipitation activity season temperature standard deviation; PWQ is precipitation of the wettest quarter; SW is surface water seasonality; TAM is year-round temperature annual mean; TASD is year-round temperature annual standard deviation; ARH is average relative humidity; and WS is wind speed. There are NAs among temperature variables as there was only one set included in each model (e.g., the *Ae. albopictus* model included PhotoASTM and PhotoASTD but not TAM, TASD, PrecipASTM, or PrecipASTD).

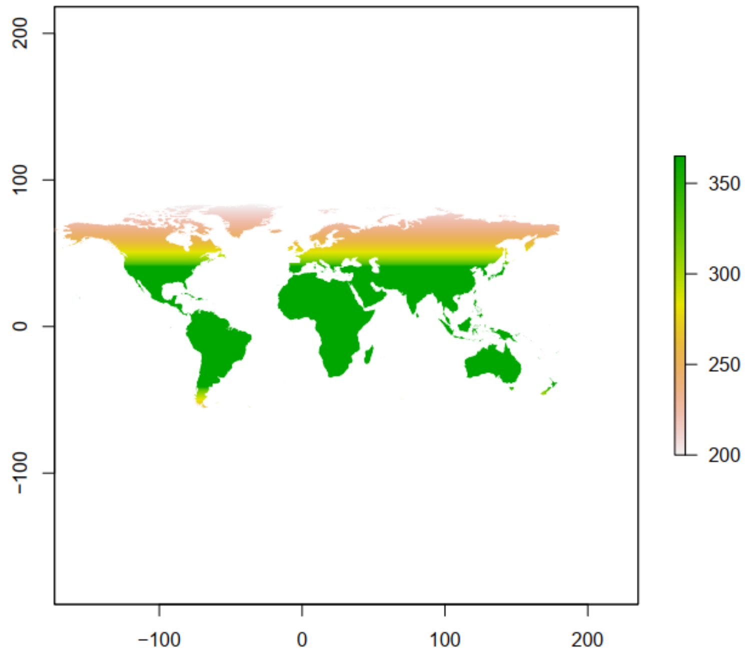

Figure B. Photoperiod activity season. Map of the world shaded in with the length of the photoperiod activity season.

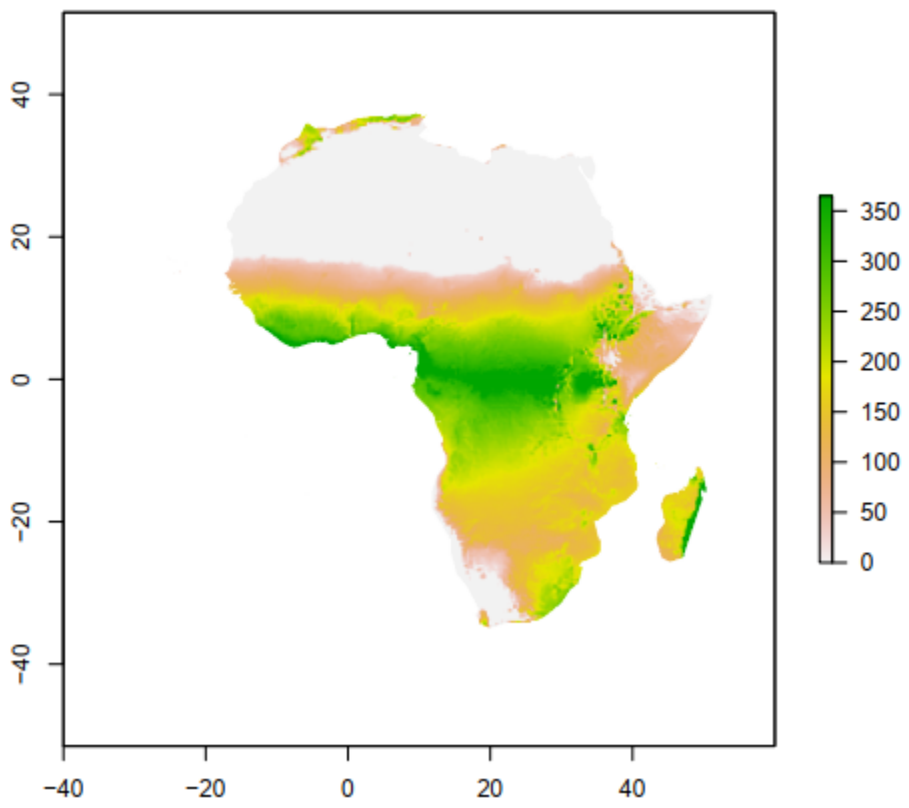

Figure C. Precipitation activity season. Map of Africa shaded in with the length of the precipitation activity season in days. Source for precipitation data is described in Table B.

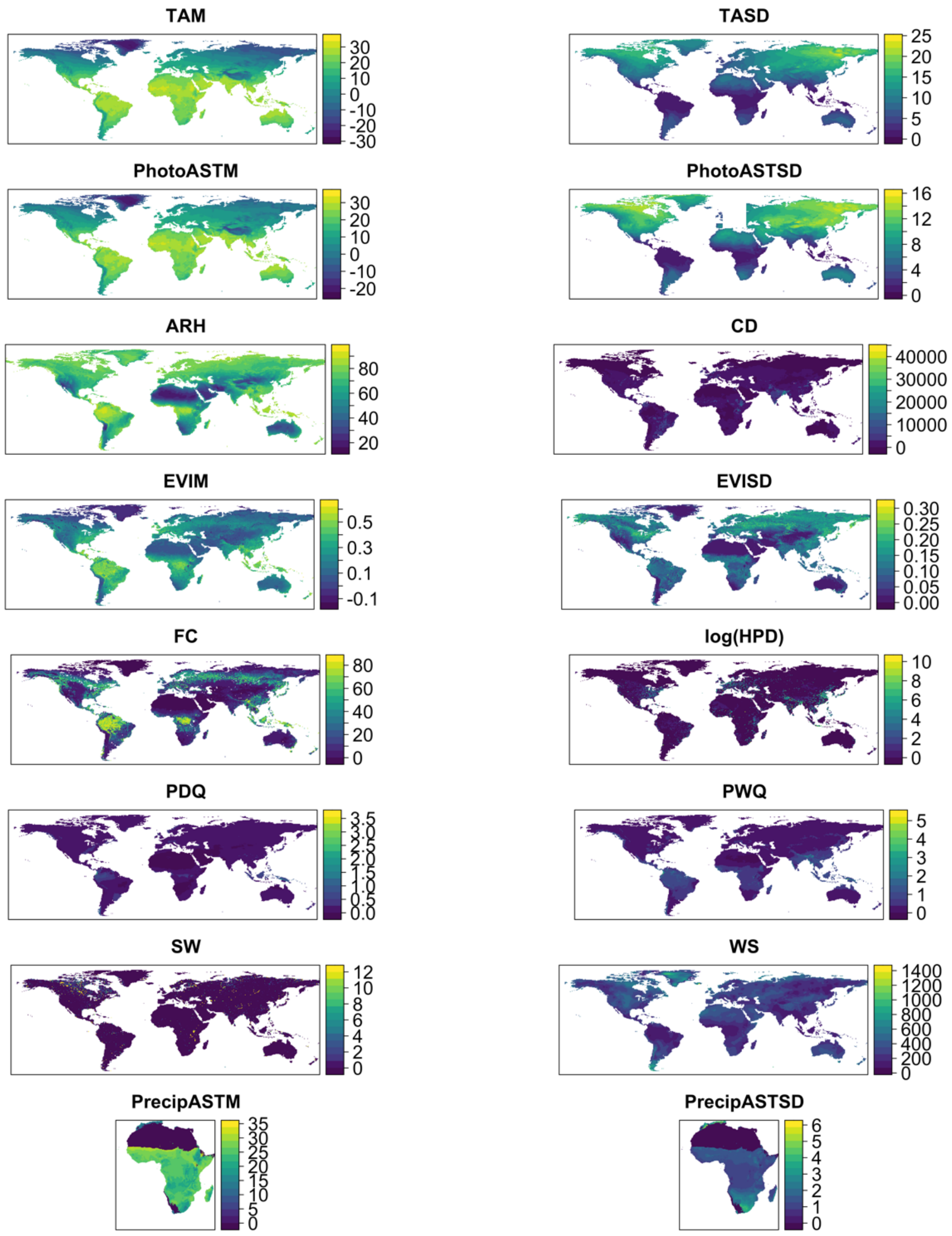

Figure D. Raster plots of all environmental covariates used in the model. Geographic and spatial distribution of every covariate used in the model, including cattle density (CD), enhanced vegetation index mean (EVIM), enhanced vegetation index standard deviation (EVISD), forest cover percentage (FC), human population density (HPD), precipitation of the driest quarter (PDQ), photoperiod activity season temperature mean (PhotoASTM), photoperiod activity season temperature standard deviation (PhotoASTSD), precipitation activity season temperature mean (PrecipASTM), precipitation activity season temperature standard deviation (PrecipASTSD), precipitation of the wettest quarter (PWQ), surface water seasonality (SW), year-round temperature annual mean (TAM), year-round temperature annual standard deviation (TASD), average relative humidity (ARH), and wind speed (WS). PrecipASTM and PrecipASTSD are only depicted over Africa as this is the region the data was used (*An. gambiae*'s data points are restricted to Africa).

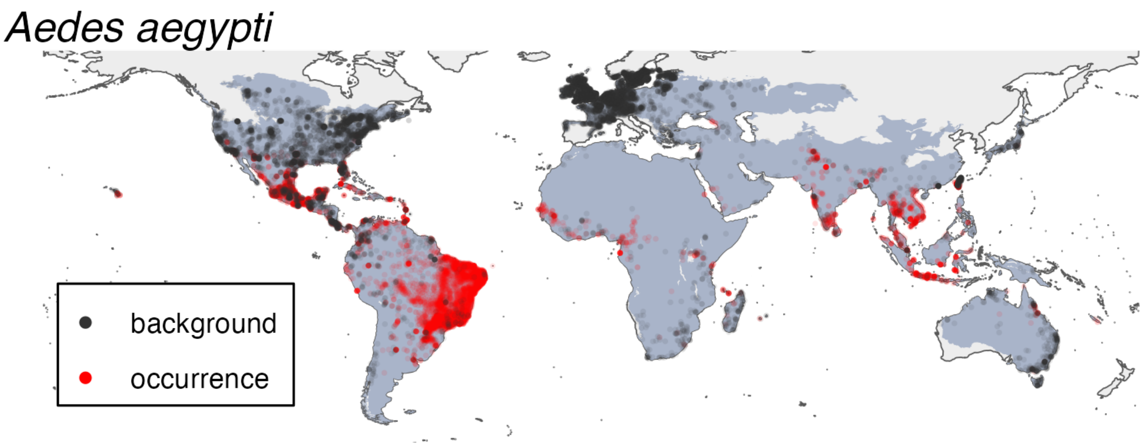

Figure E. **Species occurrence and pseudo-absence background map for *Aedes aegypti*.** Species occurrence centroids (red) and associated pseudo-absence background centroids (black) are plotted, superimposed on the respective set of ecoregions in which their buffered occurrence centroids fall and adjacent ecoregions (gray). The background shapefiles are based on from https://ecoregions.appspot.com/ and coastlines are from https://ec.europa.eu/eurostat/web/gisco.

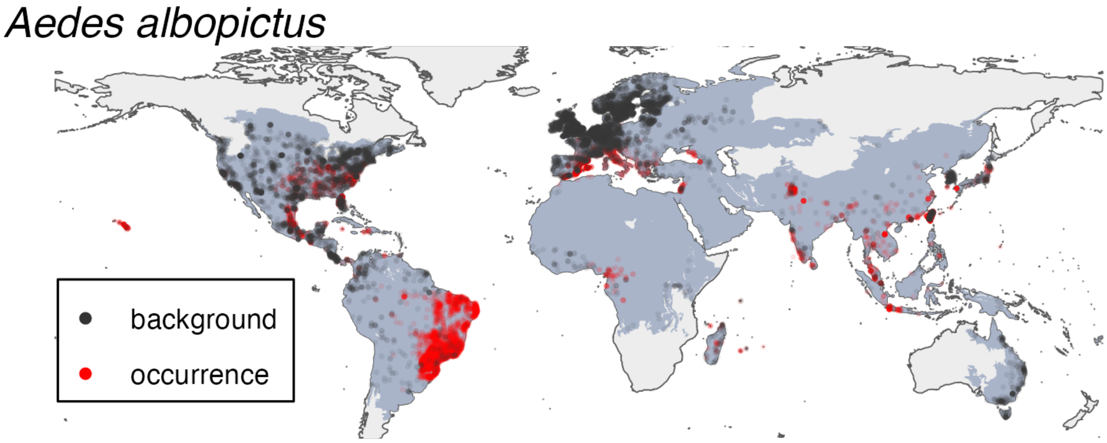

Figure F. **Species occurrence and pseudo-absence background maps for *Aedes albopictus*.** Species occurrence centroids (red) and associated pseudo-absence background centroids (black) are plotted, superimposed on the respective set of ecoregions in which their buffered occurrence centroids fall and adjacent ecoregions (gray). The background shapefiles are based on from https://ecoregions.appspot.com/ and coastlines are from https://ec.europa.eu/eurostat/web/gisco.

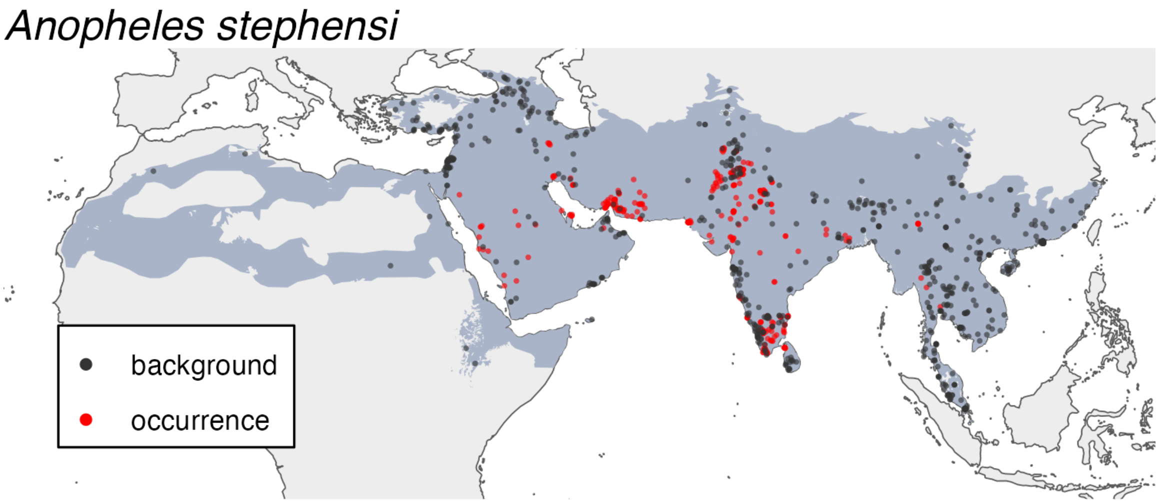

Figure G. **Species occurrence and pseudo-absence background maps for *Anopheles stephensi*.** Species occurrence centroids (red) and associated pseudo-absence background centroids (black) are plotted, superimposed on the respective set of ecoregions in which their buffered occurrence centroids fall and adjacent ecoregions (gray). The background shapefiles are based on from https://ecoregions.appspot.com/ and coastlines are from https://ec.europa.eu/eurostat/web/gisco.

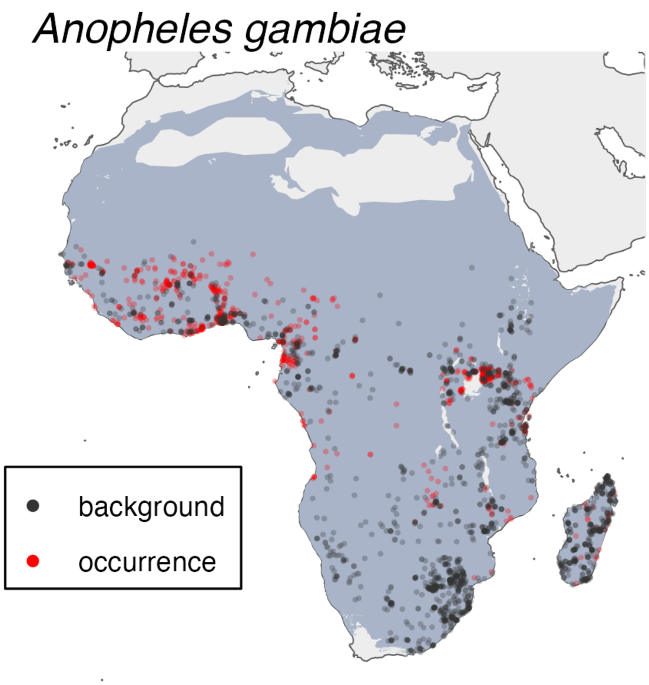

Figure H. **Species occurrence and pseudo-absence background maps for *Anopheles gambiae*.** Species occurrence centroids (red) and associated pseudo-absence background centroids (black) are plotted, superimposed on the respective set of ecoregions in which their buffered occurrence centroids fall and adjacent ecoregions (gray). The background shapefiles are based on from https://ecoregions.appspot.com/ and coastlines are from https://ec.europa.eu/eurostat/web/gisco.

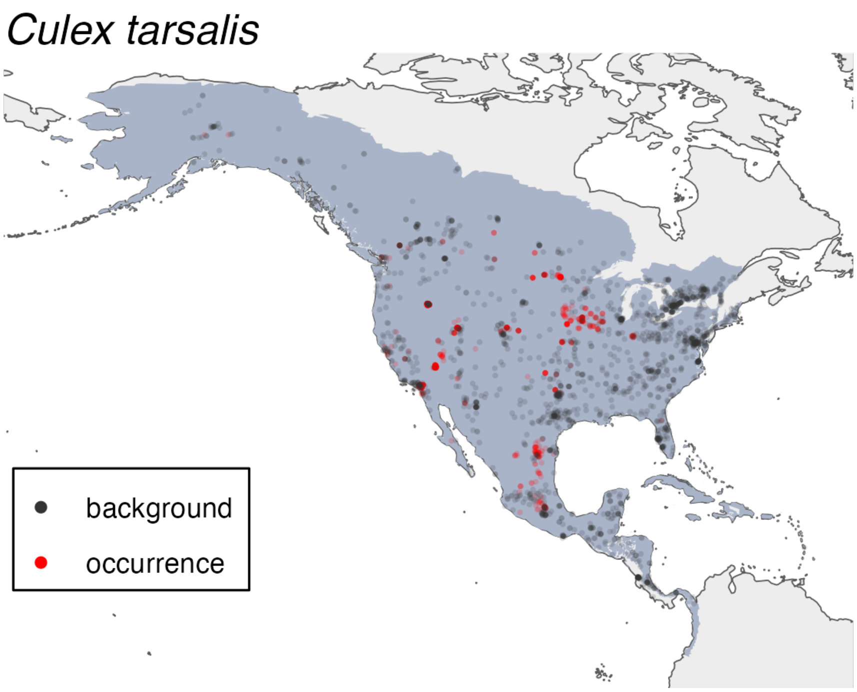

Figure I. **Species occurrence and pseudo-absence background maps for *Culex tarsalis*.** Species occurrence centroids (red) and associated pseudo-absence background centroids (black) are plotted, superimposed on the respective set of ecoregions in which their buffered occurrence centroids fall and adjacent ecoregions (gray). The background shapefiles are based on from https://ecoregions.appspot.com/ and coastlines are from https://ec.europa.eu/eurostat/web/gisco.

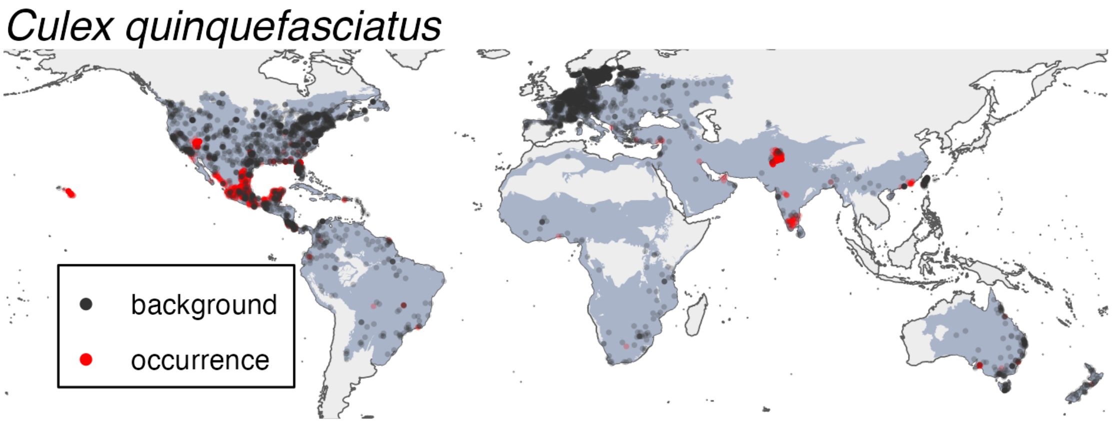

Figure J. **Species occurrence and pseudo-absence background maps for *Culex quinquefasciatus*.** Species occurrence centroids (red) and associated pseudo-absence background centroids (black) are plotted, superimposed on the respective set of ecoregions in which their buffered occurrence centroids fall and adjacent ecoregions (gray). The background shapefiles are based on from https://ecoregions.appspot.com/ and coastlines are from https://ec.europa.eu/eurostat/web/gisco.

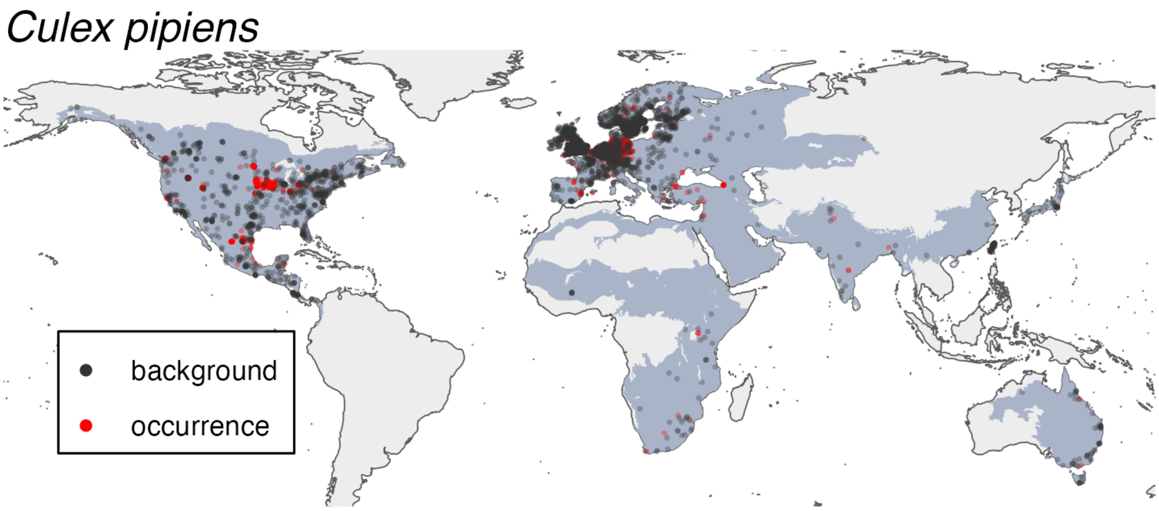

Figure K. **Species occurrence and pseudo-absence background maps for *Culex pipiens*.** Species occurrence centroids (red) and associated pseudo-absence background centroids (black) are plotted, superimposed on the respective set of ecoregions in which their buffered occurrence centroids fall and adjacent ecoregions (gray). The background shapefiles are based on from https://ecoregions.appspot.com/ and coastlines are from https://ec.europa.eu/eurostat/web/gisco.

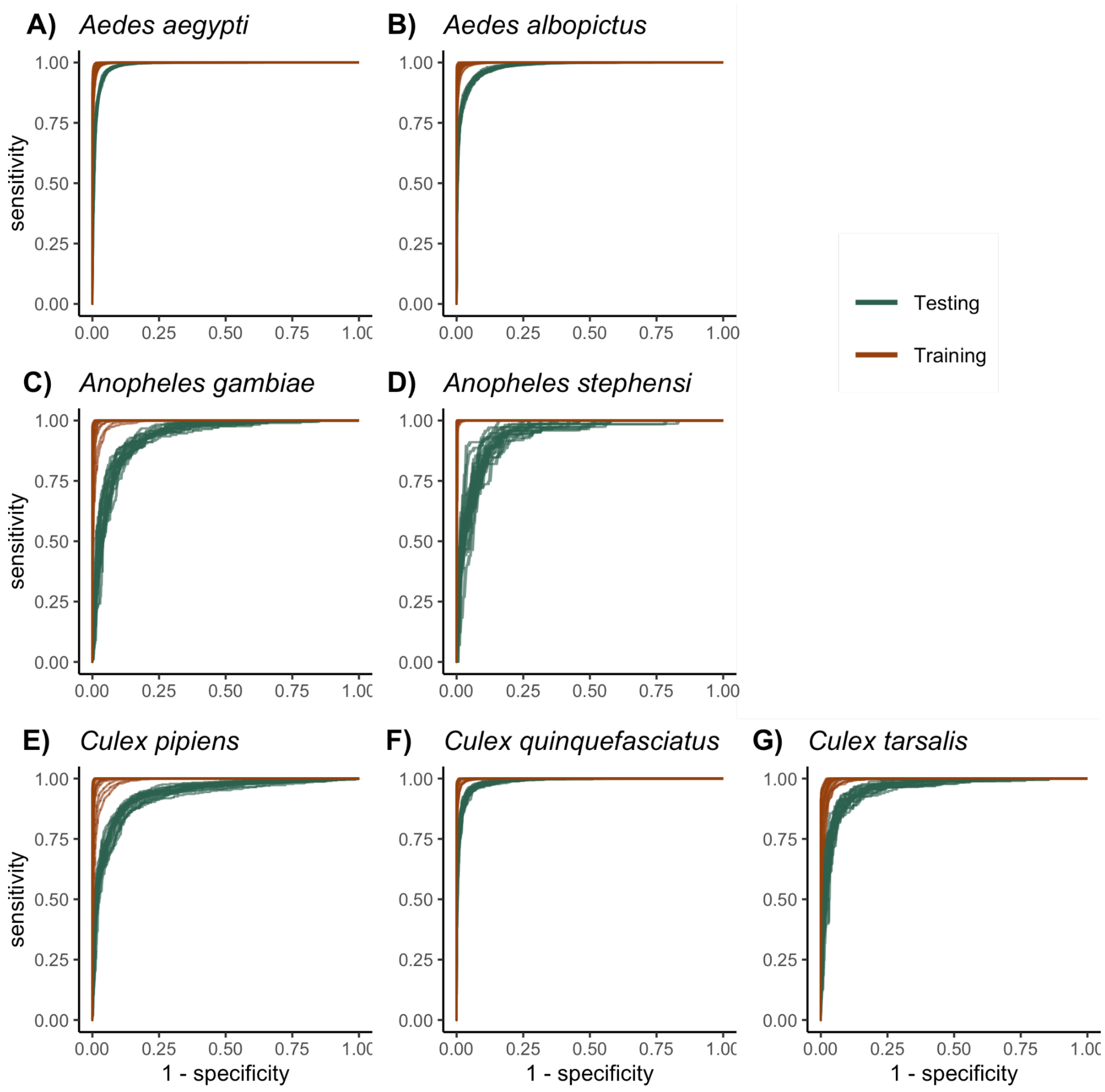

Figure L. XGBoost models accurately predicted mosquito occurrence in- and out-of-sample. Receiver operating characteristic (ROC) curves and area under the curve (AUC) values for assessment of model discrimination are depicted, where 1 represents perfect discrimination of presence and absence and 0.5 represents discrimination no better than chance. Curves depict the training set (red) and the evaluation set (blue).

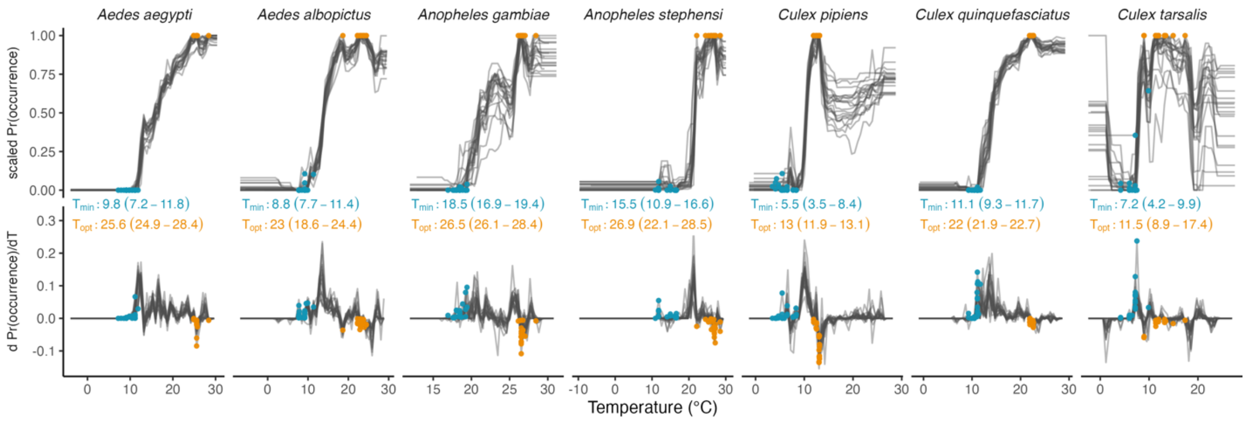

Figure M. Thermal minima and thermal optima derived from the PDPs. Thermal minima were identified as the temperature at which the partial dependence plot began increasing, which we operationalized as the first time the empirical derivative was positive and stayed positive for the next step in temperature as well (Fig. 1c). Thermal optima were identified as the point where the empirical derivative was zero and the partial dependence plot was at its maximum (Fig. 1c). We did not identify thermal maxima, as few species had partial dependence plots that clearly declined after the thermal optima and then reached a lower plateau that was within the range of observed temperatures. Central thermal minima and optima values are medians across the 20 model iterations, and parentheses indicate the full range of values over the model iterations.

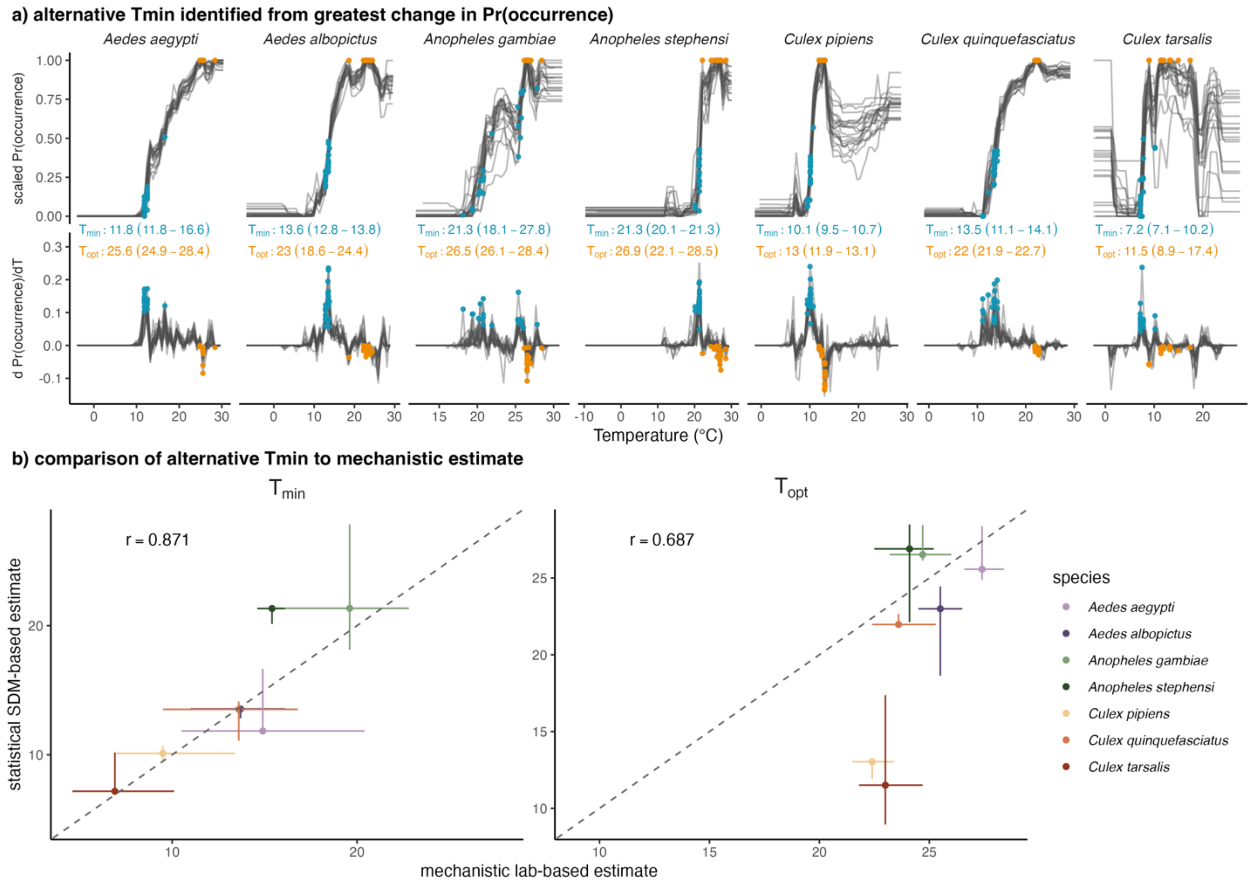

Figure N. Comparison between statistical and mechanistic under alternative definition of T_{min}. Lower thermal limits are instead identified as the temperature with the largest empirical derivative in the PDP. Panel a is as in Fig M. Panel b is as in Figure 4.

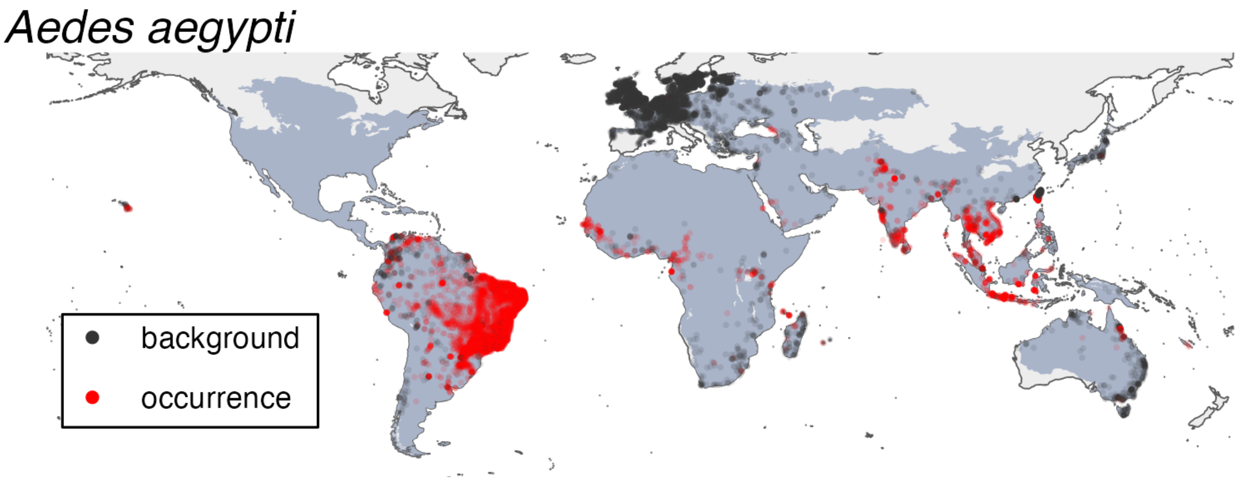

Figure O. **Species occurrence and pseudo-absence background map for *Aedes aegypti* without North America.** Species occurrence centroids (red) and associated pseudo-absence background centroids (black) are plotted, superimposed on the respective set of ecoregions in which their buffered occurrence centroids fall and adjacent ecoregions (gray). The background shapefiles are based on from https://ecoregions.appspot.com/ and coastlines are from https://ec.europa.eu/eurostat/web/gisco.

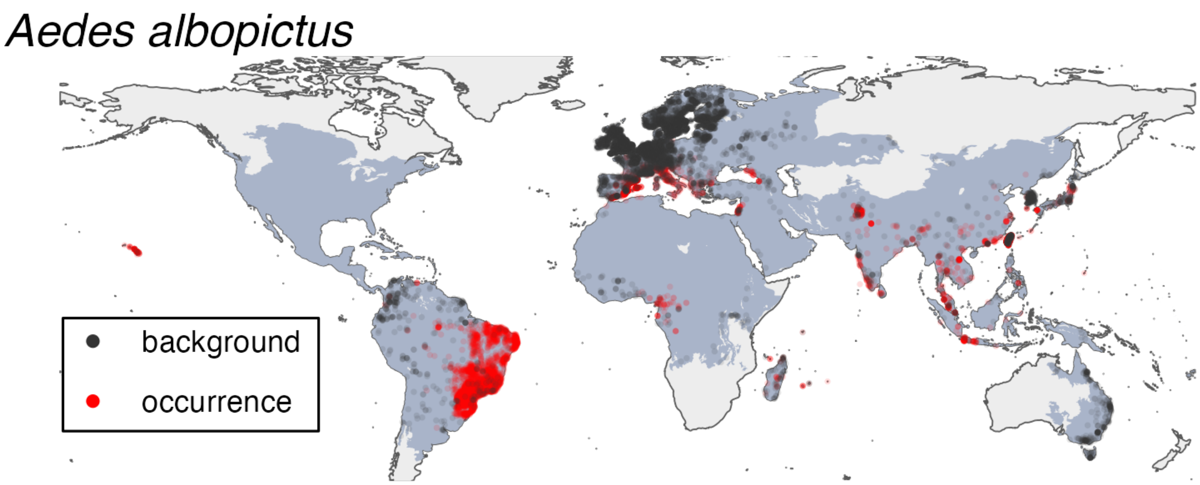

Figure P. **Species occurrence and pseudo-absence background map for *Aedes albopictus* without North America.** Species occurrence centroids (red) and associated pseudo-absence background centroids (black) are plotted, superimposed on the respective set of ecoregions in which their buffered occurrence centroids fall and adjacent ecoregions (gray). The background shapefiles are based on from https://ecoregions.appspot.com/ and coastlines are from https://ec.europa.eu/eurostat/web/gisco.

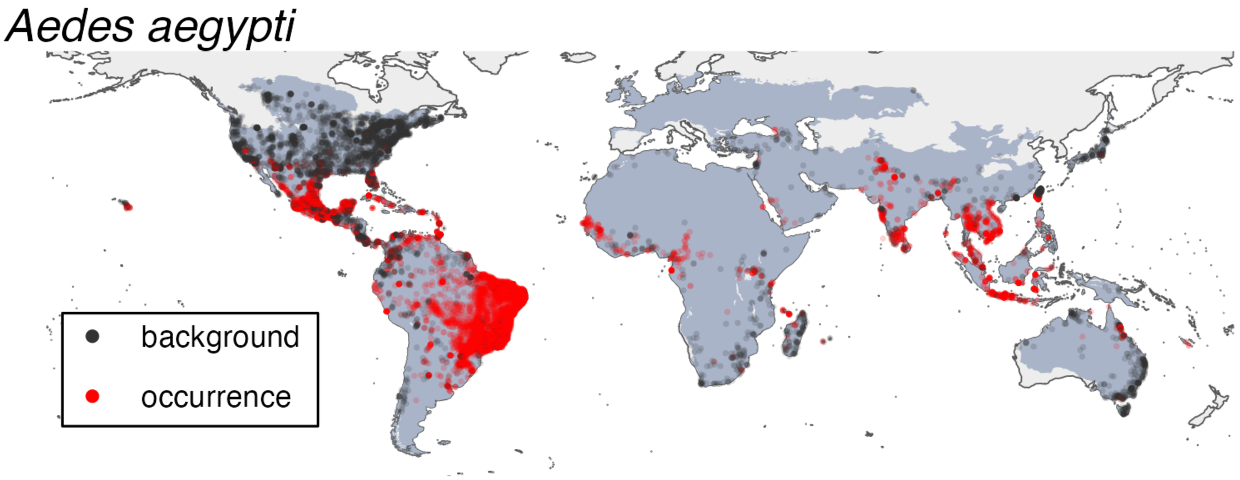

Figure Q. **Species occurrence and pseudo-absence background map for *Aedes aegypti* without Europe.** Species occurrence centroids (red) and associated pseudo-absence background centroids (black) are plotted, superimposed on the respective set of ecoregions in which their buffered occurrence centroids fall and adjacent ecoregions (gray). The background shapefiles are based on from https://ecoregions.appspot.com/ and coastlines are from https://ec.europa.eu/eurostat/web/gisco.

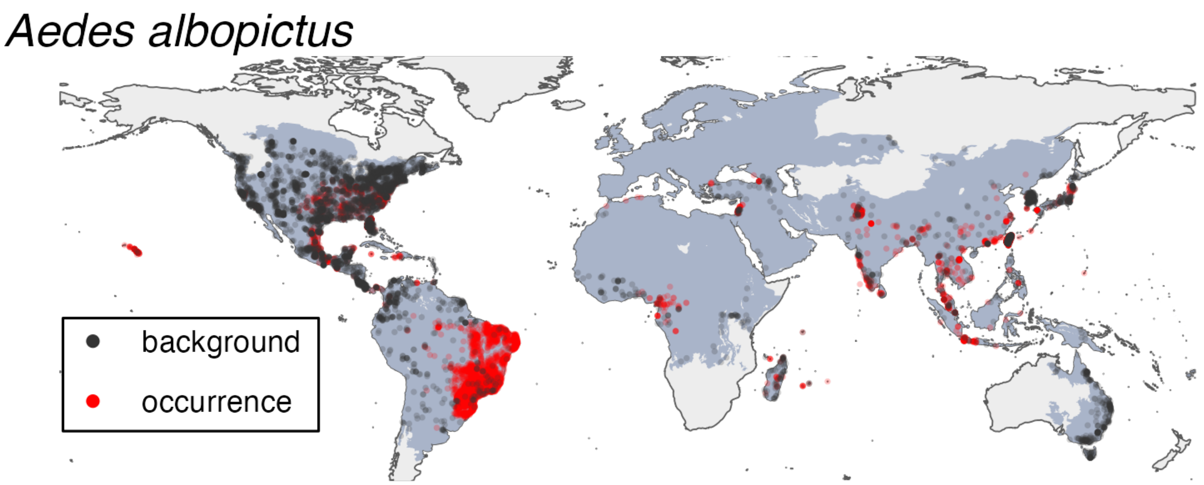

Figure R. **Species occurrence and pseudo-absence background map for *Aedes albopictus* without Europe.** Species occurrence centroids (red) and associated pseudo-absence background centroids (black) are plotted, superimposed on the respective set of ecoregions in which their buffered occurrence centroids fall and adjacent ecoregions (gray). The background shapefiles are based on from https://ecoregions.appspot.com/ and coastlines are from https://ec.europa.eu/eurostat/web/gisco.

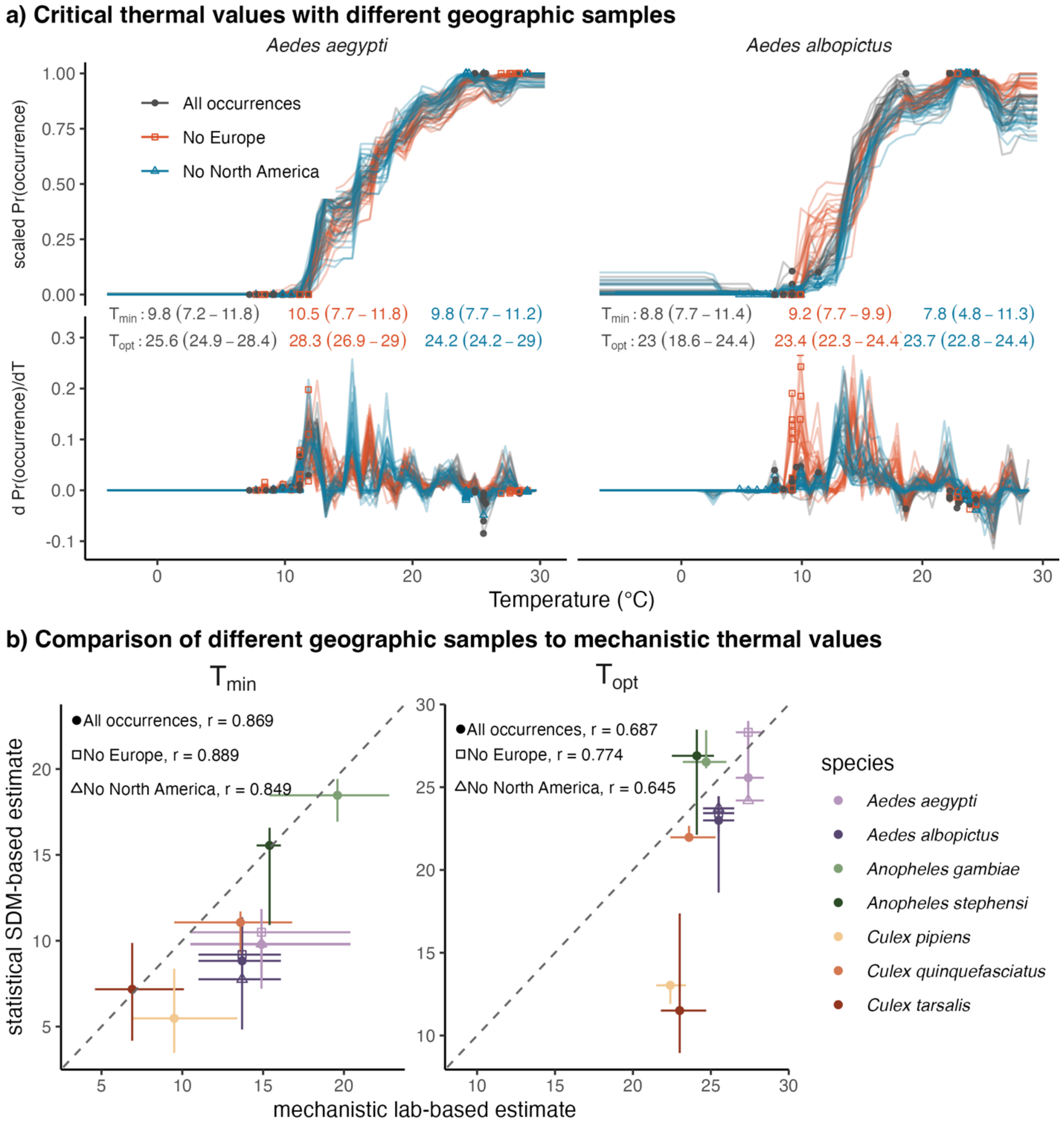

Figure S. Critical thermal minima and optima identified with different geographic samples. a) Scaled probability of occurrence (top row) and the derivative of probability of occurrence (bottom row) from partial dependence plots for the full sample (grey lines and circles), without Europe (red lines and squares) and without North America (blue lines and triangles). Text labels between panels indicates the median, minimum, and maximum for the thermal minima (T_{min}) and thermal optima (T_{opt}) across the 20 model iterations. b) As in Fig. 4, but showing T_{min} (left panel) and T_{opt} (right panel) from the three different geographic samples (all occurrences [circles], without Europe [squares], and without North America [triangles]). Each panel displays the Pearson's correlation for the different geographic samples.
