# Supplementary material for: Temperature dependence of mosquitoes: Comparing mechanistic and machine learning approaches
Source: PLoS Negl Trop Dis. 2024 Sep 16;18(9):e0012488. doi: 10.1371/journal.pntd.0012488 (PMC11460681; doi:10.1371/journal.pntd.0012488)
Supplement: Attachment — Submitted filename: mosquito_thermal_SDM_reviews_response.pdf [file pntd.0012488.s003.pdf]

Dear Dr. Glidden,

Thank you very much for submitting your manuscript "Temperature dependence of mosquitoes: comparing mechanistic and machine learning approaches" for consideration at PLOS Neglected Tropical Diseases. As with all papers reviewed by the journal, your manuscript was reviewed by members of the editorial board and by several independent reviewers. In light of the reviews (below this email), we would like to invite the resubmission of a significantly-revised version that takes into account the reviewers' comments.

Most of the reviewers have expressed major concerns and reservations on fundamental aspects of experimental design and approaches that have significant impact on your findings. These concerns must be fully addressed for your MS to be considered for publication.

We cannot make any decision about publication until we have seen the revised manuscript and your response to the reviewers' comments. Your revised manuscript is also likely to be sent to reviewers for further evaluation.

When you are ready to resubmit, please upload the following:

[1] A letter containing a detailed list of your responses to the review comments and a description of the changes you have made in the manuscript. Please note while forming your response, if your article is accepted, you may have the opportunity to make the peer review history publicly available. The record will include editor decision letters (with reviews) and your responses to reviewer comments. If eligible, we will contact you to opt in or out.

[2] Two versions of the revised manuscript: one with either highlights or tracked changes denoting where the text has been changed; the other a clean version (uploaded as the manuscript file).

Important additional instructions are given below your reviewer comments.

Please prepare and submit your revised manuscript within 60 days. If you anticipate any delay, please let us know the expected resubmission date by replying to this email. Please note that revised manuscripts received after the 60-day due date may require evaluation and peer review similar to newly submitted manuscripts.

Thank you again for your submission. We hope that our editorial process has been constructive so far, and we welcome your feedback at any time. Please don't hesitate to contact us if you have any questions or comments.

Sincerely,

Paul O. Mireji, PhD
Section Editor
PLOS Neglected Tropical Diseases

Paul Mireji
Section Editor
PLOS Neglected Tropical Diseases

****

Most of the reviewers have expressed major concerns and reservations on fundamental aspects of experimental design and approaches that have significant impact on your findings. These concerns must be fully addressed for your MS to be considered for publication.

Reviewer's Responses to Questions

Key Review Criteria Required for Acceptance?</br></br>
As you describe the new analyses required for acceptance, please consider the following:</br></br>

Methods</br></br>
-Are the objectives of the study clearly articulated with a clear testable hypothesis stated?</br>
-Is the study design appropriate to address the stated objectives?</br>
-Is the population clearly described and appropriate for the hypothesis being tested?</br>
-Is the sample size sufficient to ensure adequate power to address the hypothesis being tested?</br>
-Were correct statistical analysis used to support conclusions?</br>
-Are there concerns about ethical or regulatory requirements being met?</br></br>

Reviewer #1: Review comments.

Manuscript PNTD-23-01598 tiled: “Temperature dependence of mosquitoes: comparing mechanistic and machine learning.
This study discusses the increasing global public health concern posed by mosquito vectors (e.g., Aedes, Anopheles, Culex spp.), which transmit diseases like dengue, Zika, chikungunya, West Nile, and malaria. The authors argues that mosquitoes are shifting geographically due to climate change and other human activities. As ectotherms, mosquitoes are highly sensitive to temperature, affecting their life history traits (like biting rate and survival probability), which show upper and lower thermal limits and intermediate optima in lab studies. According to the authors, the correlation between lab-based thermal responses and mosquitoes' responses in natural settings is unclear. To bridge this knowledge gap, the study used machine learning models based on thousands of global mosquito occurrences and high-resolution satellite data

to estimate vector thermal responses. This approach, which included adjustments for mosquito activity season and ecologically relevant spatial
sampling, revealed a strong correlation between laboratory-estimated thermal minima and field observations (r = 0.90), with a moderate correlation for thermal optima (r = 0.69). However, thermal maxima were not detectable in field distributions for comparison with lab estimates. The study concluded that lab studies can effectively predict lower thermal limits and optima of mosquitoes in the field. Additionally, lab-based models might capture physiological limits at high temperatures, crucial for understanding mosquito responses to climate change, which are not apparent in field observations.

Thank you for this clear summary of our findings.

First impression: The study title "Temperature dependence of mosquitoes: comparing mechanistic and machine learning. I am contemplating whether it is appropriate to draw comparisons between methodologies that fundamentally differ from each other. For instance, mechanistic models are process-driven and are typically calibrated using data derived from controlled biological experiments. These models have a clear and traceable logic in how they process information, closely following biological phenomena as observed in laboratory settings. In contrast, machine learning models often function as 'black boxes.' Their internal workings in processing data are not transparent, making it challenging to understand precisely how they arrive at their outputs. Furthermore, these models, primarily developed from extensive datasets, may lack a direct linkage to biological or ecological principles. They are designed to identify patterns and make predictions based on the data they are fed,
without necessarily incorporating the underlying biological or ecological mechanisms. Therefore, comparing these two types of models might overlook the inherent differences in their approaches, purposes, and the nature of the data they are based on. While each has its strengths, they operate on different premises – mechanistic models with a focus on process and understanding, and machine learning models with an emphasis on pattern recognition and prediction.

Thank you for this comment. We appreciate this concern, and agree that the underlying assumptions of mechanistic and machine learning models differ dramatically. However, the fundamental question of our study—how comparable are temperature response relationships inferred from mechanistic vs. ML models?—stems from this exact concern, and thus this is where we believe the strength of our study lies. Despite their lack of mechanism and transparency, machine learning approaches are often used to understand the current distributional limits on species, and even their potential responses to global change. From mechanistic thermal biology studies (in simplified, laboratory environments), we know that organisms have non-monotonic responses to temperature that place constraints at both lower and upper temperature ranges, yet we do not yet know whether these constraints are captured in ML approaches. Conversely, the temperature constraints inferred from mechanistic modeling and experiments may be moderated by potentially complex interactions with other environmental factors, so their

relevance in real ecosystems also remains unknown. Thus, our goal was to compare the two approaches, which has not been done in the past and is currently unexplored in the literature, to help us bridge the gap between these varied methodologies and understand the relative strengths of each for future research. As such, the aim of our manuscript was not to show that we should replace one model with the other, but to show how these models complement each other. By doing so we can determine how to use the models simultaneously in order to gain a more comprehensive understanding of species response to global change. We have now included additional text in the Introduction (lines 78-81, 104-128) that addresses this concern.

Critical comments on the methodology used.
While the study delves into a compelling and potentially significant area of research, I have reservations regarding its methodology. In the case of mosquito studies, laboratory experiments are conducted to replicate the biophysical mechanisms defining and characterizing mosquito species. These experiments aim to understand species' responses to environmental factors like temperature, focusing on the process rather than being purely data-driven. The models or equations developed to estimate the lower and upper limits of a species' developmental stages are process-bound and characteristic of each species, adhering to principles that define them. However, translating these laboratory findings to natural settings using machine learning (ML) models presents significant challenges. Discrepancies between ML predictions and laboratory findings, particularly at lower thermal optima and upper limits, can arise from various factors. The complexity of natural environments, with factors like microclimates and ecological interactions, may not be fully captured in lab settings. ML models, despite their power, depend on the quality and range of input data, which might not comprehensively represent natural conditions. Laboratory studies often simplify complex biophysical mechanisms for practicality, potentially leading to gaps when applying these rules to real-world scenarios. Generalizing lab findings to field conditions via ML might fail to account for the nuanced dynamics of mosquito ecology. Therefore, while ML holds promise in bridging lab and field studies, its application needs careful consideration and calibration, respecting the complexities of ecosystems and the inherent limitations of lab experiments and ML algorithms.

This is an excellent summary of the advantages and disadvantages of each approach, and precisely the reason for investigating the relationship between temperature responses inferred from each method.

On a smaller, technical note, since ML results are based on the data that they are fed, we performed out-of-sample tests with a bootstrapping approach to make sure the results are consistent beyond a specific training dataset. This helps to test if the model is only learning patterns of the specific data fed into the model or if the model is learning more general biological patterns that are not unique to the training data. If the estimated temperature responses had large differences between bootstrapped samples, it would suggest the patterns detected by the ML may be an artifact of the data and not the

ecological system; instead, we find that the temperature responses are largely consistent between samples (Fig 3).

Translating laboratory findings to natural settings using machine learning (ML) models can be challenging.
This is exemplified by the varying correlation levels between ML predictions and laboratory findings, particularly regarding lower thermal optima and upper limits (thermal minima and field observations showing a high correlation of r = 0.90, but a more moderate correlation for thermal optima at r = 0.69). Several factors contribute to this discrepancy:
i. Complexity of natural environments: Mosquito habitats in nature are far more complex and varied than those in laboratory settings. Factors such as microclimates, ecological interactions, and geographical diversity significantly influence mosquito behavior and survival. These elements are often not fully replicated or captured in controlled laboratory environments.
ii. Limitations of machine learning models: ML models are highly dependent on the quality and range of input data. If laboratory data do not encompass the full spectrum of natural conditions or omit essential environmental variables, these models may fall short in accurately predicting real-world scenarios.
iii. Biophysical Mechanism Simplification: For practicality, laboratory studies often simplify the complex biophysical mechanisms of mosquito species. This necessary simplification for in-depth study can create gaps when ML models attempt to apply these rules to the more intricate conditions of the real world.
iv. Generalization from Laboratory to Field: While laboratory studies are crucial for grasping the basic biology of mosquitoes, extending these findings to field conditions via ML can potentially miss the subtle and dynamic aspects of mosquito ecology in nature.
Although ML offers a valuable means to connect laboratory research and field observations, its application should be thoughtfully considered. This involves recognizing the intricacies of natural ecosystems and the inherent limitations of both laboratory methodologies and ML algorithms.

We agree with the reviewer's comments, especially those regarding the complexity of natural environments and difficulty in generalizing findings from lab to field. In the current literature, there is often a disconnect between lab-based experimental work and real-world contexts which makes it difficult to translate lab/process-based findings into estimated impacts outside of the lab. Our study aims to understand when and where these lab-based and real-world contexts differ, therefore allowing us to better contextualize lab findings. We highlight this aim in the Introduction (lines 67 - 73, 102 - 120). We address the reviewer's concerns about discrepancies between the correlation for thermal minima and thermal optima in more detail below.

Further, we only included species in the study if the available occurrence points covered a large portion of known range and so that data contained adequate variation in relation to the variation vectors experience in natural conditions (but see below for how we tested for sampling bias in Europe and America). The continued advancement of geospatial

environmental data is making it more and more possible to include essential predictor variables. We carefully selected our variables based on what we consider to be important environmental drivers and have included more details on the justification for their inclusion in the methods (lines 278-327). Additionally, we performed the analysis at a resolution reflective of mosquito biology and dispersal (1km^2 at the equator). We acknowledge that our input data still faces some of the limitations the reviewer describes, but we believe our dataset is comprehensive enough to capture real biological patterns.

Reviewer #2: The study elucidates to develop a model based on newer tools, on the occurrence of 7 important vector species belonging to Culicidae, in relation to temperature globally. However, the species distribution data used for the modelling study relies on GBIF, which remains still as a baseline data to describe the occurrence of these species globally. The authors could have chosen curated different Country wise data available in the literature. Even though the authors aim the study to be a global one, they mostly restrict the data to American (and to a lesser extent African) Countries. Vector borne diseases are mainly a problem of tropical countries, which remains the worst affected. Species occurrence data used in the study (Fig. 1a) in highly affected country by the disease, where the species they concentrate is mostly Asian and African Countries, where data on the occurrence of these species is shown as meagre.

Thank you for bringing up this point; we agree that the distribution of occurrence data in GBIF is heavily weighted towards Europe and North America (see Figures S5 - S11). This is primarily an issue for species of interest whose ranges are global (i.e., *Aedes aegypti* and *Aedes albopictus*) and less of a concern for regional species (i.e., *Anopheles stephensi, Anopheles gambiae, Culex tarsalis*). We also searched the literature for global occurrence databases for each of the species of interest and included additional observations for *Anopheles gambiae* (Wiebe et al., 2017) and *Anopheles stephensi* (Sinka et al., 2020), including 2,106 raw occurrences for *An. gambiae* and 1,126 raw occurrences for *An. stephensi*, given that these two species had low sample sizes from the GBIF database alone (see lines 336-342 in the main text describing these additional occurrence points). While more targeted data collection for each of the species of interest in African and Asian countries would better balance the sample of occurrence points, this would create additional problems for sampling background points. We currently use non-target species in GBIF to construct a background sampling effort to address the geographically biased sampling. If we were to introduce country-specific occurrence datasets, the species in GBIF would no longer serve as an accurate measure of sampling bias for the dataset, requiring additional occurrence data for non-target species to complement each of the country-specific target species datasets. While this is not feasible, we agree that with the current distribution of data heavily weighted towards some regions, and if there is regional variation in species thermal dependence, the estimates will be over-weighted towards North America and Europe. To address this, we have added a new analysis that removes occurrence and background points in North America and Europe for global species showing heavy bias towards these continents (*Ae. aegypti* and *Ae. albopictus*) to

understand whether estimates of critical thermal limits and maxima that are more representative of Asia and Africa differ from those previously found. We find in these new analyses that the thermal minima and optima estimates are robust whether we consider all occurrence points, without Europe, and without North America (Figure S19). We now explain this additional sensitivity analysis in the Methods (lines 406- 414) and Results (lines 654-658).

In addition, the parameter chosen for this modelling is only a single environmental parameter, temperature. Even though it remains crucial in mosquito survival and development, other very important parameter relative humidity which is also a very crucial one is somehow not included at all. Earlier authors, used to compute a parameter saturation deficit (a combination of both temperature and relative humidity) as both these parameters have been determined as the most important environmental parameters affecting mosquito survival and thereby their distribution.

We thank the reviewer for this concern, and agree that other parameters like humidity may have a large impact on mosquito distributions. As saturation deficit is intended to combine temperature and humidity while the intent of this study was to understand the thermal dependence of mosquitoes in the field in comparison with lab studies, we instead have newly added relative humidity as an additional covariate given recent literature on its potential importance (Brown *et al.* 2023 *Ecology Letters*). We find that the estimated thermal minima and optima change very little, and have updated all analyses through the manuscripts to include relative humidity as an additional variable.

In page 5 authors emphasis on the selection of the period of distribution as "active season". Active season is there only in temperate regions, in in tropical countries. Hope this is a global investigation. - May be modified

For this study, we define mosquito activity seasons by photoperiod (days with over 9 hours of sunlight), precipitation (days with at least 50 mm of precipitation in the last 30 days), or year round, depending on the mosquito species and previous literature suggesting constraints on mosquito activity (see Methods lines 210-225 and Table 1) to better capture the temperatures experienced by mosquitoes while they were active. The length of these activity seasons differs regionally (Figs. S2 and S3), with more tropical regions having year round activity seasons even when limited by photoperiod (due to daylength always exceeding 9 hours in the tropics) and only higher latitude areas having shorter activity seasons (Fig. S2). We now clarify this in the manuscript (lines 253 -254 216).

Reviewer #3: Methods are generally valid but need additional information. In particular, I would suggest the authors to provide more details regarding the XGBoost. One thing that is not clear to me is that how the XGboost infers the relationship between the probability of occurrence and temperature. It may be helpful if the authors can provide sort of schematic plots to help readers who are less familiar with that particular method.

To quantify the effect of temperature in our ML models we utilize partial dependence plots, which graphically depict the marginal effect of a variable on the model predictions. In this case, the partial dependence plot for temperature is calculated as the average predicted probability of species occurrence for a given temperature value across all combinations of other variables observed in the data (Goldstein et al. 2015). This approach is common in explainable AI to understand how one or more features affect the predicted outcome. We have updated the explanation in the Methods to help better clarify how this approach works (lines 374-378).

Goldstein, Alex, et al. "Peeking inside the black box: Visualizing statistical learning with plots of individual conditional expectation." Journal of Computational and Graphical Statistics 24.1 (2015): 44-65.

Results</br></br>
-Does the analysis presented match the analysis plan?</br>
-Are the results clearly and completely presented?</br>
-Are the figures (Tables, Images) of sufficient quality for clarity?</br></br>

Reviewer #1: The confusion in this study arises from the observed discrepancy in accurately predicting thermal minima and thermal optima for the same mosquito species. It is perplexing how the machine learning models can predict one variable (thermal minima) with high accuracy (r = 0.90) but show less precision (r = 0.69) in predicting the other (thermal optima), despite both being characteristics of the same species. From a biophysical standpoint, this inconsistency seems counterintuitive since both limits are integral traits of the species, influenced by similar biological processes. The fact that these thermal characteristics, both resulting from and driven by the same biological processes, show different levels of predictability challenges the logical coherence of the study's findings. This inconsistency raises questions about the underlying methodologies or data used in the study, suggesting a need for a more nuanced approach that considers the interconnected nature of these biophysical traits.

Because as species distribution models aim to predict the probability of occurrence rather than mosquito abundance, we actually find the greater predictability of thermal minima (now r = 0.869 after the addition of relative humidity to the models) compared to thermal optima (now r = 0.687) to be unsurprising. This is because once temperatures exceed the necessary threshold for mosquito populations to survive, we would expect the probability of occurrence (as estimated in an SDM) to plateau and have only minimal further increases as temperature approaches the optimal temperature for mosquito population sizes. To better isolate thermal optima, a model that predicts mosquito abundance (and therefore would need reliable abundance data) would likely be necessary. We now better explain why this result is consistent with expectations

regarding how SDMs detect the influence of environmental variables in the Results and Discussion (lines 486-492, 558-567).

Reviewer #2: Analysis had been carried out for the data they have chosen for the study. However, this is not comprehensive.
We appreciate that the dataset used for occurrence points likely misses some data not registered in global databases, but as discussed in our previous response, inclusion of additional datasets for all countries introduces additional challenges due to the need to correct for sampling bias. We more directly estimate whether the parameters of interest are sensitive to our geographic sample by dropping occurrences in North America and Europe (new Figs. S15 - S18) and find that the thermal minima and optima detected for mosquitoes are largely consistent (new Fig S19).

Reviewer #3: Results are clearly presented.
Thank you!

Conclusions</br></br>
-Are the conclusions supported by the data presented?</br>
-Are the limitations of analysis clearly described?</br>
-Do the authors discuss how these data can be helpful to advance our understanding of the topic under study?</br>
-Is public health relevance addressed?</br></br>

Reviewer #1: Another challenge in this study also lies in the selection of covariates for building the machine learning (ML) model. The criteria or process used to choose variable 'x' is not clearly articulated, raising concerns about the foundation upon which the ML model was developed. Before delving into the complexities of an ML model, which often functions as a 'black box', it is essential to engage in what I refer to as "data exploration." This process involves a thorough examination of each variable, particularly environmental ones, to understand their individual and collective contributions to the phenomena we aim to predict. Data exploration is crucial as it helps in identifying the most relevant predictors and understanding the underlying relationships within the data. This preliminary step is vital for ensuring that the ML model is built on a solid and transparent foundation, enhancing its predictive accuracy and reliability. Without this initial exploration,
there's a risk of overlooking key variables or misinterpreting their importance, which could lead to less effective models and questionable conclusions

We appreciate the opportunity to further clarify our approach to variable selection in the ML model. We took a detailed approach to determining which environmental predictor variables to include. We first looked to the literature to understand which ecological variables were consistently utilized in past SDM models. Further, we sought to understand which variables

were found to be highly important predictors in other papers for our target mosquito species. Once we identified which predictors were consistently ranked as top predictors across papers, we applied our judgment to select a set of environmental covariates that we believed could reasonably capture underlying vector ecology and biology.

For example, in this process, we identified that human population density is a variable that may be ecologically important as it represents the fact that many mosquito species are highly human-dependent by preferring human blood-feeding, inhabiting urban or peri-urban niches, and breeding in artificial containers or pools of water in close proximity to human settlements. Indeed, human population density was the most important predictor for *Ae. aegypti* in a Pakistan model (Fatima et al., 2016), and for *Cx. pipiens* in a Middle East and North Africa model (Conley et al., 2014), and was thus included as a predictor in our models. Precipitation of the driest quarter, on the other hand, was thought to capture suitable mosquito breeding habitats in the period of less standing water during the dry season. This variable was found to be an important predictor for *An. gambiae* in a Nigeria model (Akpan et al., 2018), for *Ae. aegypti* and *Cx. pipiens* in an East Africa model (Mweya et al., 2013), and for *Ae. aegypti* in a Colombia model (Cabrera et al., 2020). Enhanced vegetation index describes the quality of vegetation features like leaf area, canopy cover, and sugar resources that may provide alternate food sources or resting sites for many species (Conley et al., 2014), and may estimate seasonal patterns in gross primary production (Waring et al., 2006). The mean of this variable was found to be an important predictor for *Cx. pipiens* in Middle East and North Africa, while the standard deviation of this variable was found to account for 44.7% of model fit in past MaxEnt SDM models of the same species (Conley et al., 2014).

In this fashion, we aimed to select a set of non-overlapping environmental predictors that had ecological explainability but also historical precedent, for use in our model. This approach to variable selection is laid out in the Methods (lines 275-327). We have visualizations of all environmental covariates in the supplement (Fig S4) and their relationships via correlation plots (Fig. S1).

Reviewer #2: Authors made an investigation to model the influence of temperature on the occurrence and distribution of 7 important species of mosquitoes. Their conclusion seems to be valid. They could arrive at therma minima and therma optima values. However they could not obtain a significant correlation for therma maxima. This could be owing to lack of including another equally important parameter, relative humidity for generating their model.
Thank you for this suggestion. We have included the additional suggested covariate of relative humidity in the model and find largely similar estimates throughout the analysis. The inability of the models to estimate the thermal maxima is likely due to limited sampling at the upper thermal limits of the species ranges and the fact that some species may not yet be limited in their distributions due to their upper thermal limits. We note this in the Discussion (lines 695-700, 702-704).

Reviewer #3: Conclusions are well justified.
Thank you!

Editorial and Data Presentation Modifications?</br>
Use this section for editorial suggestions as well as relatively minor modifications of existing data that would enhance clarity. If the only modifications needed are minor and/or editorial, you may wish to recommend “Minor Revision” or “Accept”.

Reviewer #1: Reject

Reviewer #2: There are some errors such as
1) When a species is mentioned in the manuscript for the first instance those should be written in full and not as abbreviation. Page 4 lines 100-101
2) Culex quinquefasciatius, one of the species they include in the study is the main vector for Lymphatic filariasis in tropical Countries. Authors mention it as a arbo-viral vector only. Page 4 Lines 106-107.
Thank you for pointing out these omissions. We have now corrected them.

Reviewer #3: Minor Revision

Summary and General Comments</br></br>
Use this section to provide overall comments, discuss strengths/weaknesses of the study, novelty, significance, general execution and scholarship. You may also include additional comments for the author, including concerns about dual publication, research ethics, or publication ethics. If requesting major revision, please articulate the new experiments that are needed.

Reviewer #1: This study discusses the increasing global public health concern posed by mosquito vectors (e.g., Aedes, Anopheles, Culex spp.), which transmit diseases like dengue, Zika, chikungunya, West Nile, and malaria. The authors argues that mosquitoes are shifting geographically due to climate change and other human activities. As ectotherms, mosquitoes are highly sensitive to temperature, affecting their life history traits (like biting rate and survival probability), which show upper and lower thermal limits and intermediate optima in lab studies. According to the authors, the correlation between lab-based thermal responses and mosquitoes' responses in natural settings is unclear. To bridge this knowledge gap, the study used machine learning models based on thousands of global mosquito occurrences and high-resolution satellite data to estimate vector thermal responses. This approach, which included adjustments for mosquito activity season and ecologically relevant

spatial sampling, revealed a strong correlation between laboratory-estimated thermal minima and field observations (r = 0.90), with a moderate correlation for thermal optima (r = 0.69). However, thermal maxima were not detectable in field distributions for comparison with lab estimates. The study concluded that lab studies can effectively predict lower thermal limits and optima of mosquitoes in the field. Additionally, lab-based models might capture physiological limits at high temperatures, crucial for understanding mosquito responses to climate change, which are not apparent in field observations.First impression: The study title “Temperature dependence of mosquitoes: comparing mechanistic and machine learning. I am contemplating whether it is appropriate to draw comparisons between methodologies that fundamentally differ from each other. For instance, mechanistic models are process-driven and are typically calibrated using data derived from controlled biological
experiments. These models have a clear and traceable logic in how they process information, closely following biological phenomena as observed in laboratory settings. In contrast, machine learning models often function as 'black boxes.' Their internal workings in processing data are not transparent, making it challenging to understand precisely how they arrive at their outputs. Furthermore, these models, primarily developed from extensive datasets, may lack a direct linkage to biological or ecological principles. They are designed to identify patterns and make predictions based on the data they are fed, without necessarily incorporating the underlying biological or ecological mechanisms. Therefore, comparing these two types of models might overlook the inherent differences in their approaches, purposes, and the nature of the data they are based on. While each has its strengths, they operate on different premises – mechanistic models with a focus on process and understanding,
and machine learning models with an emphasis on pattern recognition and prediction.

While lab-based models and machine learning-based models do have fundamentally different approaches and premises, in this context, they aim to understand the same important phenomena. We agree that machine learning models are primarily designed to identify patterns based on large datasets, and in this case, we can provide datasets and covariates formulated to detect the same biologically relevant critical thermal values that are identified from lab-based studies so we can understand the relevance of those lab-based estimates to real world contexts. Throughout the analysis, we emphasized this particular aim, from our selection of variables to our estimation of thermal minima and optima in partial dependence plots. As a result, while the original intent of ML models may have been a “black box” for prediction, our analysis emphasizes the way in which the interpretable ML can be used to understand phenomena in addition to prediction, and the importance of careful consideration of the strengths, weaknesses, and underlying premises of both approaches. We now better clarify this in the manuscript (lines 110-128). Importantly, because ML models are often used in the literature to identify current temperature constraints on species with implications for responses to future warming, our work serves to test the validity of this approach and to highlight important thermal constraints that ML approaches may miss. Further, our analysis is not intended to prove that we should replace one method over the other, but shows that ML models and thermal performance curves are highly complementary and using them in concert

can help to gain a more comprehensive understanding of species response to global change. We better emphasize this in the abstract and introduction (lines 35-38, 78-81).

Reviewer #2: In summary, if the authors would have used a curated data on the occurrence and distribution of the concerned species as well as if they would have included relative humidity, in addition to temperature, into the environmental parameters, they could have come out with a more reliable model on the influence of climatic and environmental parameters on the distribution of these species.

Thank you for these suggestions. We have implemented the suggestion to include relative humidity and find that for some species it is among the most important predictors, and find that despite that, in the updated models there is little change in the identified critical thermal values from the PDPs and therefore little change in the overall correlation between SDM-based and lab-based thermal values. Second, while we have already added additional curated data beyond GBIF for some species and inclusion of further occurrences would introduce additional complications related to background point sampling (see above), we agree completely that there may be geographic variation in the species' thermal responses that is masked by the larger number of occurrences in North America and Europe. To understand the effect this may have, we conducted sensitivity analyses and found that the results are largely consistent across different geographic samples (new Fig. S19).

Reviewer #3: This is a clearly written paper but additional info on the method would be helpful.
Thank you for suggesting this, we have added additional clarification on the methods in lines 374-378.

PLOS authors have the option to publish the peer review history of their article (what does this mean?). If published, this will include your full peer review and any attached files.

If you choose "no", your identity will remain anonymous but your review may still be made public.

Do you want your identity to be public for this peer review? For information about this choice, including consent withdrawal, please see our Privacy Policy.

Reviewer #1: No

Reviewer #2: Yes: DR N PRADEEP KUMAR

Reviewer #3: No
